# Disparities in Emergency Department Naloxone and Buprenorphine Initiation

**DOI:** 10.5811/westjem.58636

**Published:** 2023-06-30

**Authors:** Joan Papp, Charles Emerman

**Affiliations:** MetroHealth Campus of Case Western Reserve University, Department of Emergency Medicine, Cleveland, Ohio

## Abstract

**Introduction:**

Prescribing of buprenorphine and naloxone in the emergency department (ED) has been shown to be an effective intervention. The purpose of this study was to determine the frequency of prescribing of naloxone and buprenorphine and the sub-groups that may be more or less likely to receive treatment.

**Methods:**

We used a national electronic health record database to identify patients with opioid poisoning or overdose presenting between January 2019–December 2021. Patients who were prescribed naloxone or buprenorphine were identified in this dataset and then further segmented based on self-identified gender, age, racial and ethnic identity, income categories, and social vulnerability index in order to identify sub-groups that may be less likely to be prescribed treatment.

**Results:**

We found 74,004 patients in the database whom we identified as presenting to the ED with an opioid poisoning or overdose. Overall, 22.8% were discharged with a prescription for naloxone, while 0.9% of patients were discharged with buprenorphine products. Patients were less likely to receive naloxone prescriptions if they were female, White or Pacific Islander, non-Hispanic, not between the ages of 18–65, and non-English speaking. We found the same pattern for buprenorphine prescriptions except that the results were not significant for ethnicity and English-speaking.

**Conclusion:**

Despite evidence supporting its use, buprenorphine is not prescribed from the ED in a substantial proportion of patients. Naloxone is prescribed to a higher percentage, although still a minority of patients receive it. Some sub-groups are disadvantaged in the prescribing of these products. Further study may assist in improving the prescribing of these therapies.

## INTRODUCTION

Since the onset of the COVID-19 pandemic in 2020, the opioid overdose crisis has worsened with over 100,000 deaths in the United States between May 2020–April 2021, a grim 28.5% increase from the prior year.^1^,^2^,^3^ The emergency department (ED) is a vital entry point into the healthcare system for patients seeking care for opioid use disorder (OUD) and its associated complications and offers a critical opportunity to provide lifesaving interventions such as medication to treat opioid use disorder (MOUD) and take-home naloxone.

Highly effective medications approved by the US Food and Drug Administration to treat and reduce the harmful effects of OUD and illicit opioid use are available but underused, and they are often inaccessible to people who use opioids. The MOUD with buprenorphine or methadone is evidence-based, well tolerated, retains people in treatment, reduces illicit opioid use at high doses, and reduces mortality.^4^,^5^,^6^,^7^ Treatment for OUD initiated in the ED is feasible, leads to decreased illicit drug use and increased long-term retention and engagement when paired with follow-up addiction treatment services.^8^,^9^ Despite an abundance of evidence, less than 35% of adults with OUD received MOUD treatment in 2019 across all treatment settings.^10^

Take-home naloxone is another critical tool to reduce overdose mortality and requires minimal training to be used effectively.^11^,^12^ Despite supporting evidence, studies indicate that take-home naloxone access is poor, with fewer than half of overdose patients receiving a prescription for the medication within one healthcare system and only one of 13 patients receiving a prescription nationally.^13^,^14^ Some of the reported barriers include cost, patient receptiveness, regulatory barriers, and healthcare staff time.^15^

A recent prior study has indicated that nationally, following non-fatal overdose in the ED, only one of 12 patients are initiated on buprenorphine.^14^ That study found differences in prescribing based on gender, age, geographic location, and insurance status. The database used in that study under-represented the uninsured and Medicaid patients, and there were limitations in the ability to study some subsets of patients. Our purpose in this study was to evaluate whether disparities exist for racial and ethnic minorities in naloxone and buprenorphine prescribing for patients presenting to the ED with an opioid overdose.

## METHODS

The Azure Cosmos database (Microsoft Corporation, Redmond WA) is composed of submissions from organizations that participate in the Epic electronic health record system (Epic Systems Corporation, Verona, WA). Participating organizations contribute a HIPAA-defined, limited dataset for all patients into the database. Individuals from participating organizations can obtain a user license to query the database. At the time of this study, there were approximately 130,000,000 unique patients in the Cosmos dataset. The states with the highest populations include California, Texas, Illinois, Ohio, and New York. The database does not allow the identification of the specific organizations, or the characteristics of the organizations, associated with individual patients.

We used the encounter data model to query the database. Patients with an encounter type that included an ED visit without a hospital admission were selected. Patients who expired during the ED encounter were eliminated. We further categorized encounters to select those with a Systematized Nomenclature of Medicine (SNOMED) diagnosis of heroin, methadone, morphine, and fentanyl or opioid poisoning or overdose. The SNOMED is the coding terminology platform maintained by the International Health Terminology Standards Organization. These diagnoses were chosen based on an analysis of diagnoses associated with ED naloxone administration. We identified the prescribing of MOUD for naloxone or buprenorphine using the prescribing field within the database.

The Cosmos dataset includes self-identified gender, self-declared racial and ethnic identities, and age among other demographic parameters. In this database you cannot determine the exact patient age; therefore, we used various age ranges. The database contains a social vulnerability index (SVI) based on census tract data with a higher index indicating greater vulnerability.^16^ The SVI includes components of estimated income, age, disability, household composition, minority status, language, housing, and transportation. We have reported our data as the absolute numbers and frequency. Because the database maintains HIPPA compliance any numbers less than 10 are reported as 10. Statistical comparisons were made using the chi-square test using a *P*<0.05 to indicate significance. The data is reported with the *P-*value. Our institutional review board (IRB) determined that the study did not require IRB approval as it uses a HIPPA-compliant limited dataset for analysis.

**Population Health Research Capsule**

What do we already know about this issue?

*Buprenorphine is effective at reducing illicit opioid use and increases treatment retention when initiated in the emergency department*.

What was the research question?

*Among patients who present to the emergency department (ED) with an opioid overdose, do racial and ethnic disparities exist for prescribing naloxone and buprenorphine?*

What was the major finding of the study?

*These treatments are not often prescribed in the ED. Prescribing disparities are small, likely due to greater access to DEA-waivered clinicians in racially diverse ZIP codes*.

How does this improve population health?

*Disparities in buprenorphine and naloxone prescribing may be reduced with greater overall access to these treatments in the ED*.

## RESULTS

There were approximately 53 million ED visits contained within the Cosmos database during the study period. Of these, 74,004 met the entry criteria based on the SNOMED criteria. Patients were classified as follows: 67% were male, 76% White, and 18% Black; 95% were between the ages of 18–65; and 96% reported themselves to be English speakers.

The proportion of patients prescribed naloxone by self-reported gender, age, race, ethnicity and social vulnerability index (SVI) are reported in Table 1. We found that that 22.8% of patients received a prescription for naloxone following an ED visit for opioid poisoning or overdose. Female patients were less likely to receive a prescription compared to males. Patients classified as White and Pacific Islander were least likely to receive naloxone prescriptions. Patients classified as non-Hispanic were least likely to receive prescriptions as were patients below the age of 18 and above the age of 65, along with non-English speakers. Although there were differences in prescribing based on the SVI it did not follow a consistent pattern.

Buprenorphine prescribing by subgroup is reported in Table 2. We found very low prescribing rates for buprenorphine overall at only 0.9%. Like our findings for naloxone, the groups least likely to receive buprenorphine were women and White patients; however, unlike naloxone prescribing, patients <18 and >65 were slightly more likely to receive a prescription for buprenorphine. The numbers for patients <18, however, were low and estimated due to the constraints of the database. There were inconsistent results based on the SVI.

## DISCUSSION

The opioid overdose epidemic that started in the mid-1990s with prescription medications has progressively become more lethal as illicit substances have replaced pharmaceuticals. The face of a typical opioid user has also changed with this shift to impact a more racially diverse population. In the years preceding the COVID-19 pandemic, mortality was highest among White males; however, new data suggests this is changing. There was a 48.8% increase in overdose fatalities among Blacks compared to a 26.3% increase for Whites between 2019–2020.^17^ Connecticut was the first state to report that the overdose fatality rate for Blacks had outpaced the rate for Whites in 2019; and in 2020 for the first time in over two decades the US saw an overdose death rate in Blacks that outpaced Whites by 16.3% as the COVID-19 pandemic pummeled the US.^17^,^18^

All-cause mortality in the 12 months after a nonfatal opioid overdose is 4.7 deaths per 100-person years.^7^ Following an ED overdose, among patients on any MOUD the odds of a subsequent overdose death is reduced by 30%, and length of stay is significantly reduced.^19^ Buprenorphine is associated with a decrease in all-cause mortality and opioid-related mortality.^7^ Providing MOUD in the ED environment is feasible, although both real and perceived implementation barriers exist including state prescribing laws, stigma related to treating patients with addiction, insurance restrictions and reimbursement, time constraints for busy clinicians, and timely follow-up.^20^,^21^ The US Drug Enforcement Administration (DEA) DATA-2000 waiver requirement and buprenorphine-prescribing limits posed a significant barrier to treatment access during the study period. That changed in 2023 when this requirement was eliminated by the US Congress, pavig the way for improved access to buprenorphine treatment for OUD.

The absence of opioid withdrawal in the ED should not be a barrier to prescribing buprenorphine, although it is often perceived as such. Buprenorphine may be administered to patients in the ED who are in opioid withdrawal and prescribed to any patient with moderate or severe OUD along with detailed instructions for home induction when opioid withdrawal begins.^22^ Opioid withdrawal may be delayed in patients who are dependent on long-acting opioids like methadone; however, fentanyl and its analogues are the most prevalent illicit opioids leading to overdose fatality with half-lives ranging between of 0.15–7.7 hours when injected.^14^,^23^

For many who live in areas with poor access, the ED may be the only opportunity to initiate MOUD. A 2020 report by the Office of the Inspector General (OIG) found that 40% of US counties did not have a single clinician capable of providing MOUD with buprenorphine and 56% of counties with greatest need for MOUD had inadequate capacity to provide this care.^21^ When the proportion of waivered clinicians is examined by ZIP code and race and ethnicity composition, the racially diverse ZIP codes have a greater proportion of clinicians capable of providing MOUD, suggesting that availability of a waivered clinician is less of a barrier for racially diverse populations.^24^ Once initiated, treatment retention for patients on MOUD is similar for all genders, races, and ethnicities.^25^

Our study showed that White patients were less likely to receive a prescription for MOUD in the ED. This is counter to a number of other studies that have found that Blacks or other minority patients are less likely to receive MOUD across the spectrum of treatment settings.^26^ Prior research also indicated that disparities exist among racial groups’ receipt of harm reduction services and medications to treat addiction, with Whites having greater access to all treatment modalities. Medications for OUD are also less likely to be offered to Blacks and Hispanics in short-term residential treatment, and they are also less likely to complete an initial treatment episode than Whites.^27^–^28^ When the COVID-19 pandemic hit, access to MOUD and harm reduction services were disrupted among racial or ethnic minorities who self-reported that they were 8–10 times less likely to have access to clean syringes and naloxone than non-Hispanic Whites.^29^ While our results are discrepant from other studies that report on treatment across the spectrum of treatment settings, ours is the first to examine prescribing disparities in the ED setting. The differences in MOUD prescribing are small and may reflect the overall increase in opioid use and overdose rate among minorities, who are more geographically concentrated in areas with higher access to DATA-waivered clinicians.^24^

As in two earlier single-center studies, we also found an increase in naloxone prescribing for Hispanic patients. In those previous studies, Hispanic patients were more likely than non-Hispanic Blacks and non-Hispanic Whites to receive a prescription for naloxone after opioid overdose. Another study did not find a difference in the receipt of naloxone among the Hispanic population.^30^

In our study, we found inconsistent differences in treatment based on the SVI. In prior work, CA Bridge, a program of the California Public Institute of Health, found that low-income patients and those with unstable housing were more likely to accept and receive treatment.^31^,^32^ By contrast, patients with non-Medicaid insurance, stable housing, and older patients were less likely to receive treatment. The CA Bridge program attributed this to a social cognitive theory perspective; however, further study is needed to evaluate the reasons that patients with fewer resources are seemingly more likely to accept and receive treatment.^32^

We are unable to definitively determine the reason for the discrepant results between our study and prior work; however, the previous studies evaluated MOUD and naloxone access in a variety of ED and non-ED settings using different methodologies.

## LIMITATIONS

This study was dependent on the accuracy of diagnosis coding that maps to SNOMED concepts. There may be other patients with opioid overdose who were coded in a different manner, such as having altered mental status without specifying an overdose. Further, patients may have presented with an overdose although they were then coded with a related medical problem such as aspiration pneumonia or any of the other complications of opioid overdose. We looked at the prescribing of MOUD co-incident with an ED encounter. Additional patients may have had counseling during the ED encounter that led to a follow-up visit where they were provided with MOUD. This study was focused on the prescribing based on an ED visit, and we excluded patients who were admitted and who may have subsequently received naloxone or buprenorphine prescriptions.

Our data searched prescribed medications and did not include patients who may have been given naloxone kits rather than having them prescribed. Our study was not designed to evaluate that possibility. Nor did it examine prescription fill rates, which would have provided additional information regarding access to these treatments and may be adversely impacted by the patient’s healthcare coverage and SVI. Not all patients who are eligible to receive MOUD are willing to accept it. In this database study we could not determine the reason that patients were not prescribed medication as they may have been offered the medication and refused it. We did not exclude patients being sent to residential or long-term treatment facilities. It is possible that the emergency physicians may have deferred starting MOUD to the physicians in those settings, although we have no way to determine this.

Further, the nature of the Cosmos database did not allow us to determine characteristics of the treating hospitals nor whether they have addiction consult services. As a part of our review of the data we evaluated the diagnoses for all patients who received buprenorphine prescriptions from the ED and found that the vast majority were for initiation or continuation of OUD treatment. It is possible that some of the patients coded for opioid abuse also had an overdose, although we were unable to determine this. The nature of this database did not allow us to perform multiple regression analysis to look at relationships between, for example, gender, race, or age. The Cosmos database slightly over-represents both White and Black patients and under-represents the Hispanic population compared to the overall US population. Finally, no information regarding patients’ insurance status was available to analyze.

## CONCLUSION

Despite supporting evidence for their use, naloxone and buprenorphine are infrequently prescribed following an ED opioid overdose. Whites and females are least likely to receive a prescription for buprenorphine in the ED while Whites, females and Pacific Islanders are least likely to receive a naloxone prescription. Disparities exist in ED prescribing patterns for both buprenorphine and naloxone, but the disparities were small in this study and may reflect greater access to treatment in racially diverse ZIP codes and a greater willingness to accept treatment among low-income patients with unstable housing. Further study is needed to determine what factors contribute to these disparities, what interventions can be implemented to reduce them, and whether additional disparities prevent patients from filling an ED prescription. The elimination of the DEA DATA-2000 waiver in 2023 may impact these disparities and will require future study.

## Figures and Tables

**Table 1:** Prescribing of naloxone by sub-groups.

	Naloxone		No Naloxone		*P*-value
	
	Number	Percent	Number	Percent	
Female	5,152	21.9%	18,398	78.1%	*P* = .000
Male	11,716	23.3%	38,662	76.7%	
Other	21	27.6%	55	72.4%	
White	12,145	21.7%	43,806	78.3%	*P* < .000
Black	3,530	27.0%	9,522	73.0%	
American Indian	227	26.8%	620	73.2%	
Asian	95	25.4%	279	74.6%	
Other	1,895	24.3%	5,890	75.7%	
Native Hawaiian	46	20.5%	178	79.5%	
Hispanic	1,304	23.3%	4,282	76.7%	*P* = 0.02
Not Hispanic	14,718	22.7%	50,173	77.3%	
Other	867	24.6%	2,660	75.4%	
<18 years	82	10.2%	720	89.8%	*P* < .000
18–30 years	4,631	23.5%	15,117	76.5%	
30–65 years	11,545	23.0%	38,724	77.0%	
>65 years	631	19.8%	2,554	80.2%	
SVI <25th	3,006	22.9%	10,097	77.1%	*P* = 0.04
SVI 25th–50th	4,532	22.3%	15,777	77.7%	
SVI 50th–75th	6,092	21.7%	21,921	78.3%	
SVI>75th	11,349	22.4%	39,367	77.6%	
English-speaking	16,328	23.0%	54,790	77.0%	*P* = .000
Non-English speaking	551	19.6%	2,267	80.4%	

*SVI*, social vulnerability index.

**Table 2:** Prescribing of buprenorphine by sub-groups.

	Buprenorphine		No Buprenorphine		*P*-value
	
	Number	Percent	Number	Percent	
Female	171	0.3%	49,876		*P* < 00001
Male	502	2.1%	23,379	97.9%	
Other	10	11.6%	76	88.4%	
White	444	0.8%	55,507	99.2%	*P* < 00001
Black	168	1.3%	12,884	98.7%	
American Indian	10	1.2%	842	98.8%	
Asian	10	2.6%	369	97.4%	
Native Hawaiian	10	4.3%	221	95.7%	
Other	93	1.2%	7,692	98.8%	
Hispanic	63	1.1%	5,523	98.9%	*P* = .012
Not Hispanic	573	0.9%	64,318	99.1%	
Other	37	1.0%	3,490	99.0%	
<18 years	10	1.2%	802	98.8%	*P* < 0.02
18–30 years	147	0.7%	19,601	99.3%	
30–65 years	492	1.0%	49,777	99.0%	
>65 years	34	1.1%	3,151	98.9%	
SVI <25th	149	1.1%	12,954	98.9%	*P* = .00001
SVI 25th–50th	253	1.2%	20,056	98.8%	
SVI 50th–75th	279	1.0%	27,734	99.0%	
SVI>75th	676	1.3%	50,040	98.7%	
English-speaking	656	0.9%	70,462	99.1%	*P* = 0.09
Non-English speaking	17	0.6%	2,801	99.4%	

*SVI*, social vulnerability index.
